# Political science meets physical science: The shared concept of stability

**DOI:** 10.1093/pnasnexus/pgad401

**Published:** 2023-12-12

**Authors:** George W Breslauer, Kenneth J Breslauer

**Affiliations:** Department of Political Science, University of California, Berkeley, 210 Social Science Building #1950, Berkeley, CA 94720, USA; Department of Chemistry and Chemical Biology, Rutgers, The State University of New Jersey, 123 Bevier Road, Piscataway, NJ 08854, USA; The Rutgers Cancer Institute of New Jersey, 195 Little Albany St, New Brunswick, NJ 08901, USA

## Abstract

A biophysical chemist and a political scientist team up to explore striking parallels between the requisites of “stability” and the causes of instability within both the cellular/molecular world of biophysical chemistry and the world of social and political organization of self-assembled, societal structures, such as sovereign states and institutions. The structure, function, and organizational similarities of such parallelisms are particularly noteworthy, given that human agency introduces greater contingency in the sociopolitical world than do the “laws of Nature” in the natural-scientific world. In this perspective piece, we critically identify and analyze these parallels between the natural and the social realms through the prism of the shared concept of stability, including causal factors that embrace the full “stability spectrum” from instability to stability. This spectrum includes the crucial bridging, time-dependent, intermediate, kinetic state of “metastability.” Our analyses reveal that, in the microscopic/molecular world of the physical sciences, the thermodynamic and kinetic characterizations of the stabilities and transformations between physiochemical “states” exhibit cognate properties and features in the macroscopic world of sociopolitical arenas in ways that reflect a greater than traditionally assumed continuity between Nature and society. Select examples from the natural and social realms are presented and elaborated on to illustrate these parallelisms, while underscoring the striking similarities in their functional consequences.

## Introduction and background

Efforts to employ theories and methods derived from the natural and physical sciences to explain or predict social and political phenomena has a long and uneven history. John Stuart Mill, in the mid-1800s, asserted that “the backward state of the moral sciences can only be remedied by applying to them the methods of the physical sciences.” At a similar time, Karl Marx predicted that “natural science will in time subsume the science of man just as the science of man will subsume natural science: there will be one science.” In the early 20th century, Harvard's William Munro (1) proclaimed, in “Physics and Politics-An Old Analogy Revised,” that “It is to the natural sciences that we may most profitably turn …for suggestions as to the reconstruction of our postulates and methods” [(2), and references cited therein].

Despite such exaggerated “physics envy” and the unlikelihood of there ever emerging a credible, “unified theory” of natural and social life, it remains worthwhile to seek parallels between the natural and the social realms and to speculate on their implications. Within this context, our purpose in this “perspective piece” is to delineate organizational, conceptual, and functional parallels between the microscopic worlds of the physical/natural sciences and the macroscopic worlds of self-assembled, organized, societal structures, such as sovereign states and institutions.

We are not the first to venture such comparisons. Simon (3) pioneered the field of “complexity theory” in biological and social-organizational evolution. Padgett and Powell (4) published seminal work on the emergence of organizations and markets, based on analogies with chemical processes of biological change. Daems (5) has deployed complexity theory in an effort to understand the emergence and evolution of civilizations [also, (6)].

Such investigations are motivated by analogical reasoning, which has a long history of yielding fundamental insights that enable discovery. As Bartha (7) has noted:

Analogies are widely recognized as playing an important *heuristic* role, as aids to discovery. They have been employed, in a wide variety of settings and with considerable success, to generate insight and to formulate possible solutions to problems. According to Joseph Priestley, a pioneer in chemistry and electricity, “. . . analogy is our best guide in all philosophical investigations; and all discoveries, which were not made by mere accident, have been made by the help of it.” Priestley may be over-stating the case, but there is no doubt that analogies have suggested fruitful lines of inquiry in many fields. Because of their heuristic value, analogies and analogical reasoning have been a particular focus of AI research. Hájek (2018) examines analogy as a heuristic tool in philosophy.

In this perspective piece, we describe ways in which certain characteristics and tendencies in social and natural life appear to be analogous. Much of the previous work comparing these realms has focused on emergence and change. By contrast, we seek to identify similarities in the consequences of changes in conditions and the forces and perturbations that promote the stability or instability of molecular systems and sociopolitical organizations. More specifically, and as a bridging concept, we foreground the idea of “metastability,” a term familiar to students of physics and chemistry, but perhaps less familiar to social scientists. It can be an intermediate state between instability and stability. Descriptively, metastability is a precarious, rate-dependent state of stability that is relatively long-lived. It often is described as a non-equilibrium, kinetic state of stability; one that can be destabilized or restabilized when internal or external perturbations disrupt and/or surmount the most vulnerable pillars (barrier walls) of the preexisting stability. We describe the forces and influences that dictate and/or modulate the stability, metastability, and instability of our respective objects of study. We offer suggestive parallels between the conditions and forces that promote or inhibit the stability of entities in the microscopic world of the physical/natural sciences and in the macroscopic world of sociopolitical entities. In highlighting these parallelisms, we do not attempt to present a comprehensive review of the related literature. Rather, to illustrate our points, we use select examples of scholarship from the social and natural sciences. To avoid any inference of overreach, we underscore that it is not our intent, particularly in a perspective piece, to quantitatively reformulate the profound principles of thermodynamics into a societal equation of state but rather to provide scholarly examples of the striking conceptual, organizational, and functional parallelisms between the influences on the stabilities of physical and societal states. Perhaps one day a “renaissance intellect” will use such allegoric bases sets to quantitatively reformulate thermodynamic principles into a societal equation of state. However, at this stage, such an end point is beyond the scope of the intriguing allegoric analyses we present here—analyses that reveal impressive parallelisms between the forces that influence the stabilities of the microscopic physical states and the complexities of the macroscopic societal states, with the latter made even more complex by the intervention of human agency. Perhaps the analogic analyses presented here will encourage others ultimately to build quantitatively predictive models, recognizing that the underpinnings of such theoretical constructs must be tested against observationally validated bases sets, such as reported and analyzed in this paper.

### The concept of stability

What are the conditions that create stability, whether it be absolute (e.g. “ground state”) or precarious (metastable)? Let us start with an example from the social realm. One of us, a political scientist, has compared the consequences of reforms in the Roman Catholic Church since the Second Vatican Council of 1965 with reforms in the Soviet Union since the death of Stalin in 1953 (8, 9). Dubbing both entities “sacred institutions,” that study described how such institutions seek stability by creating and preserving a fairly homogeneous environment of population and beliefs—thereby making them resistant to heterodox influences, both from within and without. Ironically, this very insularity, which temporarily provides sacred entities with some level of stability, also makes that stability precarious. When internal or external forces penetrate the geographic, social, and philosophical barriers of such institutions, their rigid homogeneity makes them lack the plasticity or resilience required to adapt to a more heterogeneous social, economic, and political landscape, thereby destabilizing them. As such, their initially perceived stability is only precarious and transitory, though some institutions, like the Roman Catholic Church (as opposed to the Soviet party-state) can retain their metastability for centuries. In the case of the Church, metastability is easier to sustain than for modern political systems. This is because the reality of human death, and the impossibility of knowing what comes after, make religious claims by the Church unfalsifiable, while the promises of modern political systems to deliver material and social security are readily verifiable.

The other of us, a biophysical chemist, notes that similar principles apply in the physical sciences. For example, within the energy landscape of a chemical system, a metastable physiochemical state can serve as an intermediate, local minimum, kinetically “trapped” energy state, distinct from the system's lowest energy, most stable, “ground” state. When such a metastable state is fully insulated/isolated from “outside” forces (akin to an insulating “adiabatic” condition in the physical sciences, in which there is no heat flow between the system and its surroundings), it can maintain a precarious metastability nearly indefinitely—just like insulated sovereign states, such as North Korea, can do for a long period of time. However, if sufficient forces from the surroundings (both internal and external) breach the isolating boundaries of either a chemical or societal system, a previously metastable entity can be perturbed, altered, and, depending on context, regionally or globally destabilized.

On both molecular and societal scales, putatively “stable” entities/domains, in whole or in part, often reflect kinetically trapped states that can be destabilized and altered by a change in conditions that causes a breach in their previously impenetrable boundaries or borders. On a molecular level, such changes can induce a redistribution of chemical species, forming an ensemble of altered states and associated lifetimes. As demonstrated by Jens Völker and KJB (10–13), as well as by Pete von Hippel and his collaborators (14, 15), such altered domains (“states”) each are themselves composed of ensembles of microstates which are in rapid, dynamic interconversions with each other as well as with other invading molecular states. These molecular events, often induced by a breach in the system's insularity and/or perturbations from within, can yield new, more heterogeneous ensembles of interrelated and interconverting states—a molecular process analogous to the destabilization, fragmentation, and rebellion instigated within previously isolated sovereign states, once their geographic, social, and philosophical borders have been penetrated.

### The complexity of the concept of stability in the natural sciences

The cornerstone of the institutional/molecular parallelism we underscore here focuses on the concept of metastability and, by extension, the overarching, general concept of “stability.” In science, stability masquerades as a “simple” concept; yet in reality, it has a great deal of complexity embedded within it (16), as elaborated below.

In the physical sciences, a *thermodynamically controlled* transformation is one which produces a product that is most stable under a given set of conditions. Such a transformation corresponds to an equilibrium process. By contrast, a *kinetically controlled* transformation is one which produces a product that is formed the fastest, which corresponds to a non-equilibrium process. Complexity and differential functionality in the overall energy landscape is produced when multiple step transformations of both thermodynamically and kinetically controlled reactions proceed in temporal windows that yield both serial and parallel phasing of the transformations, thereby mapping the overall pathway as a compromise and/or optimization between speed and stability.

Some of such complexity relates to the distinction between “stability” and “equilibrium.” Stability corresponds to the resistance to change of a static system at rest, while equilibrium refers to a system's dynamic state of balance. That dynamic state affects the system's resilience; namely, its ability to accommodate incremental changes without substantially altering the system's internal organization and overall stability. Such compensatory adjustments allow adaptations that preserve or recapture the overall/global stability of the original organizational framework. As such, the descriptive property of “resilience” can be defined as the ability of a physical and/or sociopolitical system to bounce back to a stable state after a perturbing shock. Another adaptive characteristic of a societal and/or a physical system is “susceptibility,” which reflects the tendency of a system to tip over from one equilibrium to another, thereby providing the system with an additional degree of freedom short of spiraling into chaos. As such, susceptibility refers to an equilibrium reaction or process in a dynamic balance in which the rates (not necessarily the amounts) of the forward and reverse reactions are equal. In the discussion that follows, we primarily focus on a system's stability/metastability/instability as defined by its levels of resilience, susceptibility, and resistance to destabilizing change.

Greive and von Hippel (17) provide a compelling example of equilibrium and non-equilibrium considerations in their mapping of the multistep mechanism associated with the flow/transfer of information from DNA to RNA, as the DNA is transcribed and processed into its functional RNA transcripts; the latter, which provide the blueprints for protein synthesis in all cells, and as such is a central feature of life as we know it. The overall process is referred to as “transcription regulation.”

In such biological systems, complexity derives from there being other reactions/transformations that compete with the shaping of the original status quo or “ground state.” Collectively, these competing reactions may favor a trajectory that steers the system down a different pathway. Such competing pathways constitute the bases for the distinction between a simple (isolated) equilibrium reaction and one where many competing pathways are triggered that can carry the system off in another direction during the time frame in which the initial ground state reactant is “stuck” in its metastable state. Such “dendritic” branching of competing kinetically (non-equilibrium) and thermodynamically (equilibrium) controlled transformations creates the complexity of potential outcomes that depend on timescale for both biological as well as sociopolitical systems.

In this context, all reactions are reversible if one waits long enough. Reversible refers to a reaction or process in which the reactants interact to form the products and the resulting products, in turn, react to reform the reactants, with the rates of the forward and reverse reactions being equal. These conditions cause the reactants and products to exist in a state of dynamic balance, with no net change occurring. As such, “metastable” in our context refers to a conformational (functional) state that a chemical system may get “stuck in” for lengths of time that prevent equilibrium from being established before a competing (faster) reaction preferentially carries the system off in another direction.

The relative stabilities we consider depend on the respective positions of a system within the usually complex energy landscape of any molecular entity under study. Such landscapes generally are decorated with numerous local peaks, hills, valleys, and ridges within which metastable states can be temporarily trapped as precariously stable states relative to more stable ones, including the globally most stable (lowest energy) so-called “ground” state. The higher the local walls/ridges/barriers that surround and insulate these trapped metastable states, the more resistant they are to change, even though they inevitably will ultimately transition toward a more stable state when sufficient time and/or external forces are applied to overcome the barrier walls.

In this framework, change in physical and sociopolitical systems is driven and shaped by differential (e.g. relative) stabilities, reflective of the disparity in stability of one state relative to another. In the long term, the overarching trajectory of change, absent catastrophic countervailing forces, produces a path toward enhanced stability, despite at times navigating through undulating periods of instability. Consequently, over time, both change and differential stability are inextricably interwoven, with the latter driving the former, as well as driving the outcomes of Darwinian evolution—including the evolution of the genetic code as a stability-based energy ladder (18) as well as the ultimate shaping of societal hierarchical structures driven by thermodynamic selection of the most stable organizational constructs (3).

In the broad spectrum of a system's complex energy landscape alluded to above, metastability is a non-equilibrium concept—namely, a kinetic state. In this perspective piece, we do not attempt to address the debate over the utility of equilibrium models. We are, however, influenced by “complexity theory,” particularly as it relates to dynamic adaptive systems, which posits that change comes about when systems are “far from equilibrium.” As such, we consider non-equilibrium models that exhibit precarious stabilities and varying degrees of resilience, as well as models that also incorporate equilibrium components.

## Structural versus processual foci in the social sciences

As the natural sciences seek to specify the conditions for metastability of molecular assemblies and the types of perturbations that could trigger instability and/or enhanced stability, much social science during the past 50 years similarly has attempted to combine “structural” with “processual” approaches to achieve an ability to predict the relative stabilities and instabilities of sociopolitical entities. Processual refers to the study of overall, integrated processes instead of simply their component discrete events.

Social scientists working in this vein acknowledge that outcomes can be changed due to political action by leaders or social activists who do not buy into such structural inevitabilities as impersonal social forces (e.g. “class”), resource “realities,” organizational-institutional constraints, dominant values, or other entrenched forces that may heavily bias trajectories of change over time. Those social scientists who combine structural and processual approaches instead seek to specify the strength of constraints that must be overcome if the observed regularities are to change, to identify the interventions required to stretch or overcome those constraints, and to assess the conditions under which such interventions might be forthcoming and successful. (Physical scientists seek comparable goals on physiochemical states at a molecular level; namely, to define the magnitudes of the constraints that must be overcome to alter the molecular stability of the system.)

To cite but one example in the sociopolitical realm: there is a rich literature on transitions to democracy that has accumulated during the past half-century. This literature has gotten richer and better over time, as social and political scientists observed the success or failure of more such attempted transitions and the erosion of earlier gains. These scholars used such information to update their understanding of what it takes to overcome constraints, as they iterated toward a better understanding of the forces that yield or frustrate the stabilization and consolidation of new democracies.

Thus, a focus on structural constraints and boundaries, followed by an effort to determine the circumstances under which such conditions can be altered through perturbations, internal and external, finds expression in both the natural and the social sciences, as further elaborated below.

### Metastability: maintaining political stability during periods of adaptation and reform

Metastability postulates that the near *status quo* of a system can persist (exhibit “apparent” stability) as long as it remains insulated from severe internal or external perturbations (as in North Korea for the past 78 years). However, once the system tries to adapt to a changing environment, the prospect of instability looms, as induced by changes within the system. Indeed, in the social realm, Alexis de Tocqueville wrote some 100 years ago, that the most perilous time for bad governments is when they try to reform themselves [(19) edition, p. 157].

So, to the social scientist, the question is: how can political systems adapt without substantially destabilizing themselves? Gorbachev's strategy of adaptation failed, as it eventually brought down the entire system. But Chinese leaders, while facing political challenges (such as the Tiananmen Square protests of June 1989), have managed to retain essential political stability, while still reforming their economy and partially opening themselves to the outside world—with successful socioeconomic results, albeit at the price of allowing grand-scale corruption that has become a potential Achilles heel and has rendered the system only metastable. Lee Kwan Yew of Singapore and Kemal Ataturk of Turkey were both “authoritarian modernizers” who, like China, managed to transform their economies and cultures by dictatorial means but, in contrast to China, without triggering or tolerating grand corruption.

The sacred institution of the Vatican, since the Vatican Council of 1962–1965, has proven itself able to adapt without fundamentally destabilizing itself (by schism, for example) because it has not adopted reforms as radical as those that Gorbachev advocated for the Soviet political system. Analogously, President Franklin Roosevelt managed to save the precarious capitalist system by reforming it with his New Deal policies. By contrast, the liberal government that assumed power in Russia after the abdication of the Tsar in February 1917 was unable to restabilize the political system precisely because it insisted on staying in World War I. That World War had been the perturbation that created the conditions for the February Revolution against tsarism. Staying in the war perpetuated and deepened those system-destroying conditions and made possible the Bolshevik seizure of power later that year. Thus, human interventions can lead metastability toward policies that either facilitate the retention of metastability (Roman Catholic popes since 1965, Deng Xiaoping, Franklin Roosevelt, Lee Kwan Yew, Kemal Ataturk) or that create a cascade into instability (Gorbachev, the 1917 liberal government in Russia).

### Systems approaches to political and biological entities

In the 1950s and 1960s, some leading political scientists [e.g. (20)], inspired by an urge to apply natural-scientific systems approaches to the study of social and political orders, adopted a “systems” approach to the study of politics. The emphasis was on the interrelationships among components of the “system,” the requisites of systemic equilibrium, adaptation or change, and the role of political actors in inducing such changes. In Easton's case, the political subsystem was viewed as the instigator of changes that could alter and re-equilibrate the system through political action in response to internal or external stimuli disruptive of the preexisting equilibrium.

It is noteworthy that many interdisciplinary biological scientists investigating complex biological processes also were adopting a “systems” perspective in which they abandoned the reductionist approach in favor of mapping all the numerous interactions between the components of a complex system, the so-called interactome (21). The latter dynamic constellation of numerous coupled and uncoupled functional interactions of the interactome collectively map the wiring diagram that constitutes the system as an integrated whole, rather than the simplistic reductionist approach of assuming the whole simply to be the sum of its parts. In biology, the information-rich potential of this more integrated, multicomponent approach motivated the NIH to establish a Laboratory of Systems Biology (21).

In a related approach, some political scientists embraced “structural functionalism,” which posited that structures within the political system perform certain functions that contribute to the political system's stability, during, for example, such longer-term processes as “modernization” (22). Thus, so-called “modernization theory” posited that growing complexity within society needs to be mirrored by increasing structural differentiation through which to channel that complexity. Growing functional differentiation requires equivalent structural differentiation. This expectation in sociopolitical systems for structural differentiation to scale in growth with structural complexity, as manifest in functional differentiation, is regularly mirrored in the biological sciences. For example, the evolution of organelles such as mitochondria, with their specialized structures and functions, allow cells to address the increasing complexities and functional needs of evolving cellular systems. Analogously, Mark Field (23) argued in the 1960s that the Soviet Union represented a case of “constricted political development,” precisely because all significant political action had to be channeled through the Communist Party, even as industrialization and modernization fostered growing societal and economic complexity that called for greater structural differentiation. These integrated systems perspectives were much like that being employed by the biological community to explain the functioning and stability of complex systems, which were viewed metaphorically as biological “machines” (21). On this score, one might refer to the structural-functional perspective as “mechanistic.” Additional examples of such parallels can be extended. For now it is noteworthy that such political and social responses to a range of internal and external stimuli have much in common with the complex, coordinated, multicomponent immune system responses to endogenous and exogenous foreign molecular invaders (21).

Also in the 1960s, Chalmers Johnson (24) integrated a systems approach into his theory of the causes of revolutions. There he argued that stable political systems find themselves in a state of “homeostatic equilibrium”—thus borrowing a metaphor from the physical sciences. Homeostatic equilibrium refers to the tendency of a self-regulating system to establish a relatively stable equilibrium between interdependent elements. As such, it is a self-regulating process by which a system tends to maintain stability while adjusting to conditions that are best for its survival.

As defined by Johnson, stability is not synonymous with “stasis.” Rather, the homeostatic quality of the equilibrium allows for incremental adjustments within a certain range. The system is, then, metastable—to use the characterizations employed here. But when this system is too severely disturbed, such that the barriers are overcome that protect the metastable state, a more drastic, even revolutionary situation may result.

So, what are the forces that shape such sociopolitical energy landscapes? Johnson argued that a social system's stability hinges on congruence between its “value system” and its “division of labor.” In this context, congruence refers to the quality of agreeing, being in accord, being in compliance.

Johnson proposed that when incongruence or disequilibrium takes place between these two elements (i.e. when people widely conclude that the existing division of labor in society is fundamentally unjust), the preconditions for revolutionary change are in place, requiring only a catalyst to light the spark. Analogously, Eckstein (25) developed a theory of “congruence,” according to which stabilizing governmental performance may hinge on congruence between authority relations in the political realm and authority relations within other units of society.

Even political scientists who did not explicitly adopt systems approaches often advocated for some variant of an equilibrium model to understand the requisites of political order. Thus, Samuel Huntington (26) argued that “order” requires that there be a symmetry between the level of demanded societal participation and the level of institutionalization of avenues for such participation, whether that participation is voluntary (in democratic systems) or obligatory (as in Leninist dictatorships that mobilize their citizenry into state-directed organizations). A good example of this phenomenon in a democratic system was the extension of the vote to 18-year-olds in the United States in the wake of anti-war protests of the mid- and late- 1960s. (Notably, these protests had been heavily populated by students under the age of 21.) Another example from a Leninist system has been the Chinese Communist Party's strategy of coopting new social and economic elites that emerged from its economic reform programs.

Thus, “equilibrium” theorists, who, at times, co-mingled the varied physical and chemical meanings of equilibria models in the context of political stability, generally treated the polity as a system and focused on the interrelationships and interactions within the system that they considered to be decisive for maintaining stability.

Interestingly, in the social sciences, systems approaches based on models of equilibrium largely faded away with time. They were superseded by approaches that sought to specify the interaction between policy interventions and structural constraints, as leaders and activists sought to achieve specified goals. We suggest a few examples that vary in both their geographical scope and time-scale. In a single-country study covering a three-decade period, Ang (27) deployed this approach to identify “how China escaped the poverty trap” as a result of reforms initially launched by Deng Xiaoping and elaborated by his successors. In a study of multiple countries in Latin America over the course of almost a century, Collier and Collier (28) demonstrated how the alternation of regime-types in this region was a product of cyclical interactions between fluctuating political coalitions and both internal and external socioeconomic constraints. And Lebow's (29) study was even more encompassing across time and space, exploring the rise and fall of political orders on many continents over the course of many centuries.

Likewise, those who study revolutionary change rarely embraced Johnson's model of homeostatic equilibrium. Instead, they focused on the interaction between conditions at the level of the elites and conditions at the level of society (30)—and occasionally on underlying forces that triggered changes in these immediate conditions at both levels [see, most recently and impressively, (31)]. Specifically, they conclude that, while societal alienation is necessary, it is insufficient to trigger revolutionary change. The latter also requires a widespread perception that elites (or the military and security services) are either too collectively demoralized or too internally divided to agree to repress societal defiance. Absent such a perception, social actors are prisoners of the “collective action” problem: alienation will not turn into behavioral defiance until social forces coalesce in the perception that defiance will not be easily repressed. As such, successful revolutionary action requires a widespread perception that defiance is both desirable *and* feasible. Feasibility requires both perceptions of opportunity and mechanisms for coordinating collective action. Once a perception of opportunity takes hold, and the collective-action dilemma (the coordination of defiance) is overcome, revolutionary protest suddenly accelerates. In the social science literature, this is referred to as the “cascade effect.” Consider the case of Eastern Europe in 1989, where the entire bloc of communist states collapsed within eight months. The political systems in the communist bloc appeared to remain metastable until Poland negotiated an end to communist rule in Spring 1989, which triggered reverberations through the eastern bloc, demoralizing other communist elites and emboldening other populations living under communist rule. When Hungary opened its borders with Austria, this triggered another accelerant: thousands of East German citizens flocked to fellow-socialist Hungary to take advantage of a border opening that allowed them eventually to reach West Germany. And ultimately, in November 1989, when the Berlin Wall was breached, the end to communist rule in East Germany was swift, as thousands of East Germans rapidly fled to the West, by transport or on foot. Without thinking in terms of cascade effects and accelerants, one cannot understand the speed with which seemingly metastable communism collapsed in the eastern bloc. Without thinking of those political systems as insulated entities, the borders of which were suddenly and almost simultaneously punctured by heterodox influences from without and within, one cannot understand the sudden impact of kinetic processes on the preexisting thermodynamic state.

To better appreciate the congruence between these accelerant phenomena in the natural and social sciences, a closer examination of the concept of “cooperativity” is in order. Cooperativity is a term that can refer both to macroscopic phenomena, up to and including human behavior (“collective action”), and to microscopic/molecular-scale phenomena. The term can be used to refer to the coalescence of the many degrees of freedom of a large group of people, or to a chemical macromolecule, when either entity abruptly changes states in a rapid, coordinated, concerted, manner, rather than in an incremental stepwise manner. As such, cooperative transformations are threshold phenomena that manifest in both societal action by groups of people when sufficient numbers collectively realize their power as a coordinated entity, as well as at the molecular level, when a chemical entity abruptly transforms from one state to another due to a critical level of alignment of interdependent, favorable interactions that trigger the cascade of coordinated events that yield the concerted production of the “product” state.

### The architecture of complexity

In search of the sources of stability and instability in social organization, “complexity theorists” have sought to identify the organizing logic built into organizations and institutions. Herbert Simon's article of 1962, “The Architecture of Complexity,” was seminal in this regard (3). Notably, there Simon also explored briefly the parallel between the social and the natural realms by relating the architecture of complexity to both sociopolitical organization and biological evolution. In explaining the architectures of social structures in terms of complexity, Simon wrote, “But this is nothing more than survival of the fittest—that is, of the stable.” Thus, in both realms, Simon endorsed a Darwinian perspective that privileges stability (to us, including metastability) as a causal driver. (A most-recent statement of the long-term evolution of societies, based on complexity theory, has been laid out briefly by Dries Daems ((5); see also (6)).

Analogous “thermodynamic selection” arguments have been posited by Breslauer (32) for energy-based drug design, and, as noted above, by Horst Klump, Jens Volker, and Ken Breslauer (18) to yield an energy-based, reductionist explanation for the molecular complexity associated with the evolution of the genetic code, as well as with the genotypic stability concept of “molecular Darwinism,” as an adjunct to Darwin's “survival of the fitness” phenotypic arguments.

### The reciprocal concepts of decomposition and coalescence

In his essay on the architecture of complexity, Simon also introduced the idea of “decomposition,” a phenomenon that has conceptual parallels in both the natural/physical sciences and the sociopolitical sciences (3). Decomposition refers to the breaking up of a chemical or societal entity into its constituent parts. As an organization theorist, Simon argued that if the complexity of a system is “architectural,” then there are certain principles built into the structure that determine both its architectural stability *and* the lines along which it would come apart (decompose) if it yielded to instability. Such a predictive analysis, based on the balances between overarching, stabilizing structural motifs, versus regional, structural fault lines that favor decomposition, can in the social realm be applied to federalism (politically strong center, politically strong regions—USA), confederalism (weak center, strong regions—Canada, Switzerland), regional organizations (like the European Union), empires, and any other composite entities composed of disparate units. The Soviet Union—a unitary state (strong center, weak regions)—decomposed along the lines of the ethnic republics of which it was composed, as did Yugoslavia (a federation), Czechoslovakia (a confederation) and, earlier, French Indochina (an empire).

In the natural world, one can identify analogous principles of decomposition. A chemical compound is an example of a composite entity composed of multiple component units, such as elements and chemical clusters of elements. Under a given set of conditions or influences (e.g. temperature, pressure, polarizability) a constellation of elements and chemical groups can coalesce (the conceptual opposite of Simon's decomposition) to form a compound. This coalescence to form a stable compound occurs when the structure/architecture of the resulting compound allows the net attractive intermolecular forces between the component chemical units to exceed the net repulsive forces. This mapping of the key stabilizing molecular interactions allows one reciprocally to define the loci of fault line sites, which, when perturbed by an alteration in conditions, will cause the compound to decompose, yielding chemical products of enhanced stabilities under the new conditions. However, this new chemical stability can itself be precarious, since when circumstances again change, the decomposition products of the original compound can themselves decompose to produce a new set of products that are more stable under the altered conditions. The trends in the thermally induced decomposition of the nitrates and carbonates provide impressive predictive examples of chemical behavior that mirrors the molecular fault lines of instability that can result in the dissolution of unitary chemical states, thereby leading to the fragmentation of the “compound state” to yield a multiplicity of new “chemical states” based on alterations in key stabilizing or destabilizing molecular interactions.

The chemical behaviors just noted are mirrored within the decomposition and coalescence of sovereign governmental states. In response to changes in circumstances within the world order, often based on real and/or perceived economic, military, social, and political advantages or threats, sovereign states formally and informally realign via coalescence (think NATO, the European Union) and decomposition (the Warsaw Pact, Brexit). Once the real and/or perceived advantages of either coalescence and/or decomposition no longer create a strategic advantage, the profiles of the sovereign states may remold themselves again, frequently along historical and ethnic lines. The cascade of decompositions that may ensue in the natural realm has counterparts in the political—as when, for example, collapse of the USSR into 15 sovereign states led to decomposition pressures within those new states (think Chechnya within Russia or Nagorno-Karabach within Azerbaijan). These sovereign state/societal influences can be viewed as corresponding to the key intermolecular forces associated with the stabilization/destabilization of chemical compounds that, once disrupted, define the sites of the fault lines that trigger change.

In this context, the organizational frameworks of hierarchical, societal governing structures also are mirrored in many ways by atomic and cellular structures and their associated more regional functions. The overarching influence of a centralized, federal government is mirrored by the dominating influence of the centralized atomic and/or cellular nuclei. Under the auspices of such centralized power, multiple regional governments function—a feature mirrored by the atomic electrons or the cellular organelles, with the net functional result being a compromise between centralized and decentralized forces.

Of course, the principles, influences, and forces underlying coalescence and decomposition probably are more diverse in the sociopolitical realm than in the natural world—precisely because of the plethora of political choices that inform principles of coalescence compared to the more constraining “laws” of Nature that govern the natural world. Conversely stated, the physical laws of Nature are more restrictive than the diverse influences that shape the expansive sociopolitical landscapes. Consequently, in contrast to the laws of Nature that are quantitatively derived from fundamental first principles, the “laws” and indices that govern sociopolitical entities also reflect the difficult-to-quantify influences of human agency. As a result, such indices more qualitatively score the relative changes in stability that result from the net additive balance between destabilizing increases in entropy and enthalpy-driven stabilizing increases in societal order (e.g. laws and regulations). As such, leaders or governments interested in fine tuning conditions for either stabilizing or incrementally adjusting societal norms, without risking runaway destabilization, must employ “controlled” chaos and “judicious” regulations. For example, the British colonial empire carved out nation-states in Africa, the Middle East, and South Asia, the borders of which did not correspond to then-existing ethnic and tribal divisions in the colony. This shaping of the colonies often was intentional—a means of sociopolitically playing tribes and ethnic groups off against each other (“divide and conquer”). In the wake of de-colonization, decomposition of the empire often left states without nations, which international organizations like the United Nations would validate and sustain, but which remained, in many cases, deeply divided and conflicted—often murderously so. Other colonial empires, whether overseas (French, Dutch, Portuguese) or contiguous (Ottoman, Austro-Hungarian, Russian) devised their own individual strategies for establishing or maintaining colonial control, largely unconstrained by the laws of Nature and driven primarily by sociopolitical and economic considerations. When decolonization occurred, these empires’ strategies of coalescence also influenced their paths of decomposition. But they did not fully predetermine that pathway based on physical laws, since external actors often intervened to deflect decolonization in one direction or another. Think, for example, of the Treaty of Versailles’ (1919) impact on the shaping of new European states after the collapse of the Austro-Hungarian and Ottoman empires. Examples are legion.

Padgett and Powell (4) provide additional examples of efforts to bring together the logic of chemistry and organization theory. In their book, they draw on the chemical concepts of “autocatalysis” and “hypercycles” to understand the emergence and stabilization of organizations and markets—both historically and in the present day. Autocatalysis in the social sciences leads to emergence via self-shaping/self-editing of the sociopolitical system, just as in the chemical sciences the autocatalytic properties of RNA have been proposed to result in the origins (emergence) of life on Earth. Autocatalytic refers to a process in which one of the products of the process also serves as a catalyst for its own formation in the same process. Such processes are self-regulating and self-perpetuating. Hypercycles, in both chemistry and societal shaping, have been used to map the sequential transformations (changes) that lead to the optimization of stability, both sociopolitical and physical. Intriguingly, hypercycles also have been employed to graphically map the cascade of sequential transformations dictated by differential stabilities, often referred to as thermodynamic selection. Such stability-dictated sequential transformations have been evoked to rationalize the evolutionary origins of the genetic code as a differential energy/stability code (18).

### Features of governmental forms that exhibit longevity

Levitsky and Way (33) recently demonstrated that autocratic regimes born in revolution, such as communist regimes and Iran, exhibit very long staying power. They argue that this longevity is due to the unity of the elites created by the revolutions as well as their ability to eliminate all rival elites and to atomize society. In short, such governments prevented the emergence of the preconditions for instability—namely, splits within the elites and the “snowballing” of coordinated popular defiance via a cooperative effect amongst the populace—much like the molecular cooperativity that triggers accelerated molecular transitions in the physical sciences. Within the sociopolitical realm, such concerted, cooperative defiance surmounts the collective action barrier that normally inhibits such coordinated group resistance.

Other types of regimes that also maintained long-term stability are long-consolidated democracies (e.g. the US, Canada, most of Western Europe, Costa Rica, Japan, S. Korea, Taiwan, Australia, New Zealand, India). Most of the rest of the world has been marked by either unconsolidated or only recently established democracies (e.g. much of Eastern Europe, parts of Latin America, several African and Asian countries) as well as varieties of authoritarian regimes (such as military juntas, personalistic strongman regimes, single-party regimes, etc.), which all fit into the category of metastable flirting with instability.

In the aggregate, the most intriguing observation is that long-consolidated democracies and totalitarian regimes (e.g. 78 years of North Korea) exhibit the greatest long-term stability. However, even longitudinally stable governments can transit to metastability when confronted by intractable crises, such as uncontrolled mass immigration into Europe and the US, major trade isolation that savages their economy, or when they seek, or are forced, to relax their controlling grip on society, such as occurred in the post-Stalinist phases in communist regimes or in Iran today.

### Heterogeneity, diversity, asymmetries, phase separations, and critical point behavior

Additional intriguing parallels can be identified between the physical and sociopolitical sciences, consistent with several of the cognate properties noted above. For example, heterogeneity in a physical system, and its cognate property of *diversity* of population in a society, can both make the system stronger, provided that the components are well integrated. As such, heterogeneity can increase physical stability. For example, mixtures have higher boiling points than pure liquids; imperfections or asymmetries in a structure can increase its mechanical strength; and copolymers can exhibit better material properties than homopolymers. For similar reasons, the corresponding sociopolitical feature of diversity may also facilitate formation of enriched and healthy relationships (and accomplishments) of a society; although under divisive circumstances, diversity may also create unstable societal fault lines that trigger and fuel decomposition and fragmentation of a state. As such, the impact of heterogeneity in the physical sciences and diversity in the sociopolitical sciences can, depending on circumstances and the degree of integration of that diversity, have net stabilizing or destabilizing influences. In this regard, it is worth noting that the physical process of phase separation (akin to societal decomposition) occurs at a “critical point” where the fluctuations, both molecular and societal, are maximized. This parallelism is reflective of the increasingly greater physical and/or societal turmoil that prevails just prior to the division/disunion, if not decomposition, of a physical state via phase separation or a societal state via rebellion, fragmentation, and secession.

### Going forward

The search for a metric that scores/reflects the relative stabilities of governments and the associated half-lives (time-dependence) of that stability (metastability) has been the goal of many world organizations, including the Unites States’ Institute of Peace, the World Bank's Worldwide Governance Indicators, the United Nations Development Program's Human Development Index, and the Economist Intelligence Unit's Democracy Index. Each organization defined a set of criteria which they score and combine to yield a weighted average of a country's attributes which are judged to be associated with stable governance and quality of life—which parallel our focus on elite unity/disunity and popular support/disaffection. They then use the aggregated results to rank order the world's countries in terms of these objective and subjective attributes (e.g. stable governance, quality of life). Specifically, in these rankings, they assess for each country the presence of: legitimate and effective institutions; responsive and inclusive processes; justice and reconciliations; access to essential services; and economic foundations. The temporal component to such stability may be derived by examining the historical record (several decades of previous rankings plus deeper historical research) to determine how long the governance stability on average is sustained in each country. The combination of these characteristics of metastability, in principle, allows one to construct a “metastability index” as a follow-up to the scholarly direction proposed in this perspective piece.

### Conceptualization of the stability of societal entities in terms of thermodynamic principles associated with the physical sciences

In the spirit of the continuity theme elaborated here between the physical and social sciences, one may, once armed with sufficient data density and variety, seek to build upon the aforementioned metastability index by reasoning analogously with the forces that influence chemical stability and the perturbations that induce chemical transformations. Consider the following forms of governance as the chemical system: direct democracy, a representative democracy, a parliamentary system, socialism, communism, a monarchy, an oligarchy, and an autocracy. These different forms of governance have at least one feature in common; namely, they each bring various degrees of order (decrease in entropy) to a sociopolitical entity. Absent any form of governance, the societal entity (system) quite likely would spiral into “chaos,” a property that is characterized by a high degree of entropy. Thus, governments in all forms bring some level of organization (reduction in entropy) which requires energy input (called enthalpy) to sustain themselves. Such governmental energy input into societal organization serves as a countervailing force to reduce the universally inherent trend toward increasing chaos, as reflected in Nature by the ever-expanding increase in entropy of the universe.

It is instructive, and perhaps more intuitive, to employ existing conceptual verbiage to characterize the parallelisms/analogies we identify between the societal and physical realms, as manifest in fundamental thermodynamic relationships. To this end, we provide the following conceptual examples of the stabilizing/destabilizing consequences of a few specific features/characteristics that we have elaborated on within the body of this perspective piece:

- Unity of elites, coupled with sustained repression of autonomous political organization within society (i.e. totalitarian systems) can be stabilizing for long periods.
- Alternatively, structural complexity that provides the population mechanisms for authentic political participation can be stabilizing, as can independent judicial and legislative branches.
- Societal fault lines associated with the decomposition or coalescence of a political order can be destabilizing, as can poorly integrated heterogeneity/diversity; whereas
- Homogeneity of population can enhance status quo stability.
- Alternatively, well-integrated population heterogeneity increases resilience, thereby stabilizing the status quo while enabling orderly incremental change absent revolution.

The net result of using fundamental thermodynamic concepts and relationships to combine the order-producing energy (enthalpy) of government with the countervailing natural tendency toward disorder (increase in entropy) yields the free energy term that corresponds to the stability of the system. At equilibrium, a government's enthalpically driven societal rules (e.g. laws, regulations, etc.) exactly balance the natural tendency of unregulated societies toward disorder (an increase in entropy), thereby creating a precarious state of stability, at least transiently for which the free energy at equilibrium equals zero. The resilience of a government (its longitudinal sustainability) reflects its ability to incrementally adjust to internal and external perturbations by readjusting or rebalancing the normative societal rules/regulations to create a modestly reformed state under a new equilibrium. Absent such resilience/susceptibility/adaptability on the part of government, the seeds of a potential populace rebellion can be planted, either at the ballot box or in the streets.

## Closing remarks

This perspective piece is meant to underscore conceptual, organizational, and functional parallels between the fundamental forces and processes that shape the molecular world and the macroscopic world, up to and including the complexities of social orders. As underscored by Simon in his analysis of hierarchical complexity, the insights and limits of such parallelisms need further elaboration, particularly as they relate to the concept of stability. It is our hope that this essay will stimulate further discussion of commonalities—as well as differences—associated with stabilizing and destabilizing influences that shape both the microscopic worlds of physical/natural sciences and the macroscopic worlds of organized, societal structures.

*Postscript:* Shortly after the beginning of the 20th century, Albert Einstein declared (34):

A theory is the more impressive the greater the simplicity of its premises, the more different kinds of things it relates, and the more extended its area of applicability. Therefore the deep impression that classical thermodynamics made upon me. It is the only physical theory of universal content which I am convinced will never be overthrown, within the framework of applicability of its basic concepts.

As a biophysical chemist and a political scientist, the authors consider the ultimate exemplar/test of this assertion to be the demonstration that the complexities of sociopolitical entities can be rationalized in terms of fundamental thermodynamic principles.

## Data Availability

No datasets were analyzed.
